# Hard template synthesis of metal nanowires

**DOI:** 10.3389/fchem.2014.00104

**Published:** 2014-11-17

**Authors:** Go Kawamura, Hiroyuki Muto, Atsunori Matsuda

**Affiliations:** Department of Electrical and Electronic Information Engineering, Toyohashi University of TechnologyToyohashi, Japan

**Keywords:** metal deposition, anodic aluminum oxide, mesoporous oxide, carbon nanotube, tubular pore

## Abstract

Metal nanowires (NWs) have attracted much attention because of their high electron conductivity, optical transmittance, and tunable magnetic properties. Metal NWs have been synthesized using soft templates such as surface stabilizing molecules and polymers, and hard templates such as anodic aluminum oxide, mesoporous oxide, carbon nanotubes. NWs prepared from hard templates are composites of metals and the oxide/carbon matrix. Thus, selecting appropriate elements can simplify the production of composite devices. The resulting NWs are immobilized and spatially arranged, as dictated by the ordered porous structure of the template. This avoids the NWs from aggregating, which is common for NWs prepared with soft templates in solution. Herein, the hard template synthesis of metal NWs is reviewed, and the resulting structures, properties and potential applications are discussed.

## Introduction

Metal nanowires (NWs) are typically prepared using templates, with the exception of those grown using nanoparticulate catalysts (Choi et al., 2008; Bashouti et al., 2012). Surface stabilizing molecules or polymers can be used as soft templates (Tang and Tsuji, 2010). Porous solid materials such as anodic aluminum oxide (AAO), mesoporous oxides (MOs), and carbon nanotubes (CNTs) can be used as hard templates. Metal NWs formed from soft templates are dispersed in solution, and require subsequent immobilization on matrices for many devices. NWs prepared with hard templates can be spontaneously immobilized in an ordered arrangement, because of the ordered porous structure of the template. Thus, metal NWs formed from hard templates can simplify device production. Metal NWs and the composites of which with hard templates have a wide range of applications, from transparent electrodes, metamaterials to biosensors (Hu et al., 2011; Shin et al., 2012; Proenca et al., 2013; Wang et al., 2013; Ye et al., 2014). Some of the applications take advantages of high periodicity and chemical stability of the template. Thus, synthesizing metal NWs using various templates has been widely investigated. In this mini-review, we discuss the use of AAO, MO, CNTs, and other materials as hard templates for producing metal NWs. The possible applications of metal NWs prepared by these methods are also discussed with comparison highlighting the advantages and disadvantages of various composite structures.

## AAO templates

**Figure 1:**
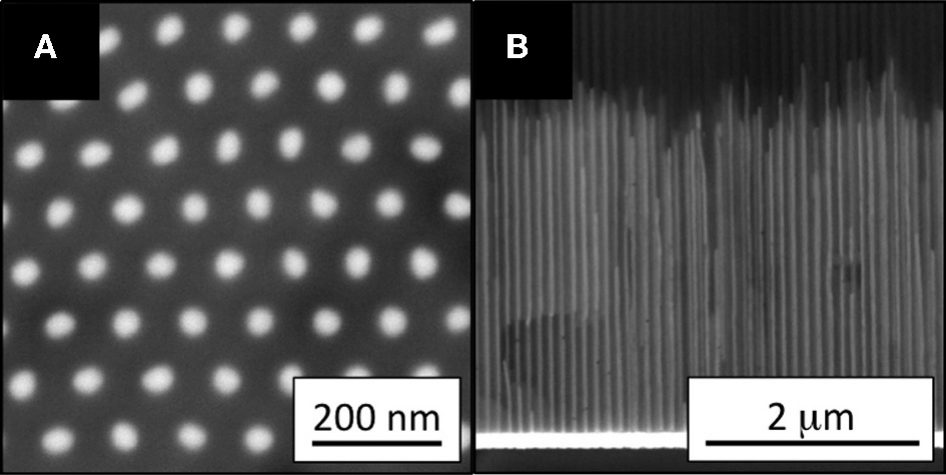
**(A)** Bottom (after milling to a depth of 200 nm) and **(B)** cross-sectional SEM images of Co NW arrays in an AAO template. The NW diameters are ~40 nm, and the interpore distances are ~100 nm. Reproduced with permission from AIP Publishing LLC (Proenca et al., 2013).

AAO contains two-dimensional hexagonal pores (Keller et al., 1953). The diameter and length of these pores can be precisely controlled by the anodization conditions, including the voltage, temperature, time and electrolyte composition (Sarkar et al., 2007; Lu and Chen, 2011; Poinern et al., 2011). The development of AAO synthesis techniques has led to the preparation of NWs with precisely controlled structures in AAO templates (Huber et al., 1994; Lu and Chen, 2011). Metal NWs have been prepared in AAO templates by casting, and vapor, supercritical fluid, chemical, electro-, electrochemical, and photochemical depositions. Electrodeposition has been most commonly employed, because of its simplicity and resulting dense NWs in high yield. Cu, Fe (Thongmee et al., 2008), Ni (Byrne et al., 2009), Co (Vivas et al., 2012a; Proenca et al., 2013), and CoNi (Vivas et al., 2012b) NWs have been prepared by electrodeposition, and their biocompatibilities and magnetic properties were investigated. All NWs except for Fe were single crystalline, without post-treatment after electrodeposition. NWs are typically synthesized in acidic solution (pH 2-5) containing H_3_BO_3_ buffer. The equipment required for electrodeposition is similar to that for anodization to prepare AAO. The AAO acts as the cathode when the metal NWs are deposited. Figure 1 shows horizontal (A) and vertical (B) cross-sectional SEM images of deposited Co NWs, with diameters of ~40 nm and lengths of ~3 μm. The horizontal cross-sectional image (Figure 1A) was captured after ion milling to a depth of 200 nm, to remove unfilled AAO template from the top of the sample. The SEM images demonstrated the homogeneity of the pore filling, and uniformity of the NWs along the pore walls. This NW uniformity allowed the detailed investigation of the effect of the NWs dimensions on their magnetic properties. In other words, the magnetic properties of metal NWs can be tailored by modifying the AAO structure.

Casting is also often used to prepare metal NWs in AAO templates. Molten metals are cast into AAO pores, without complex chemical or electrochemical processes. Thus, casting is simpler than electrodeposition. However, the high gas pressure or hydraulic force required to fill the pores with metal usually increases the production cost. The aluminum layer underlying the AAO template melts at 660°C, so the filler metal must have a lower melting point. Thus, casting is usually reported for low melting point metals such as Bi (Heremans et al., 2000), and Pd (Kuo et al., 2013). Fabricating multilayered NWs consisting of different metals, alloys, or metal compounds with distinct borders by casting is very difficult.

## MO templates

MOs, especially mesoporous silica (SBA-15 and MCM-41), have been used as hard templates for preparing metal NWs. The MO pore size is normally smaller than that of AAO, so noble metals such as Au (Kanno et al., 2012; Kawamura et al., 2012b), Pt, Ag (Han et al., 2000; Takai et al., 2010; Kim et al., 2012), and Cu (Zhang et al., 2007) have more often been used to fabricate NWs in the tubular pores of MOs. This is because of their low sensitivity to oxygen, and possible application as catalysts and components of electronic devices. The methods used to deposit the metal NWs are similar to those for AAO templates. However, the smaller pore size results in incomplete filling of template pores by vapor-phase epitaxy, chemical vapor deposition, and photochemical deposition (Leon et al., 1995; Kawamura et al., 2012a). The supercritical fluid synthesis using MOs has also been applied to base metals such as Co, and Ni NWs (Coleman et al., 2001; Holmes et al., 2003). Metal chelate precursors, M(hfa)_2_ · × H_2_O (M: metal, hfa: hexafluoroacetylacetonate) were used in this method. Supercritical CO_2_ and H_2_O have been attracting more attention as an alternative to conventional organic solvents since they are nontoxic and nonflammable.

## CNT templates

In contrast to metal NW arrays prepared from AAO and MO templates, isolated NWs can be obtained using CNT templates. Metal filling in CNTs is typically achieved by chemical vapor deposition or wet chemical processes (Tsang et al., 1994; Li et al., 1998; Govindaraj et al., 2000). One problem with this method is the low percentage of filled CNTs caused by their small inner diameter, high aspect ratio and high curvature. Metal impregnation into CNT interiors can also be achieved by incorporating metals and metal precursors with the carbon source during CNT growth. Pd, Pb, Bi, Y, Mg, Gd, Ti, Cr, Fe, Co, Zn, Mo, Ta, W, Dy, and Yb have all been trapped inside CNTs by this method (Guerret-Piecourt et al., 1994; Sen et al., 1997; Liu et al., 2000). Impurities are often produced, including encapsulated carbon clusters and soot. CNTs are not wetted by liquids with surface tensions higher than 100–200 mM M^−1^, so there have been limited reports of molten metals penetrating into CNTs by capillary forces (Ajayan and Iijima, 1993; Ugarte et al., 1996). The rapid filling of Pd, Ni, and Cu into multiwalled CNTs has reportedly produced metal/multiwalled CNT composites. Supercritical CO_2_ was used as the reaction medium, and long metal NWs were prepared (Ye et al., 2003). Au, Pt, and Pd NWs were fabricated by the templated electrodeposition of metals on the outer surface of CNTs (Dudin et al., 2011). Although the application of metal NW/CNT composites has not become real yet, they can be used as catalysts, sensors, phtothermal nanomaterials, and in electrochemical energy storage and production by taking advantage of CNT characteristics including high chemical stability (Che et al., 1999; Weissker et al., 2010; Rossella et al., 2012).

## Other templates

Au NW arrays have been prepared by electro-, chemical, electrochemical, and photochemical deposition on track-etched porous polycarbonate (TEPP) membranes, which were used as hard templates. The dimensions of this template are much larger than those of AAO and MO, so it is better suited to biosensor applications (Cusma et al., 2007; Lu et al., 2007; Yang et al., 2007).

Si is the most commonly used material in microelectronics, photonics, and sensing, so composites of metal NWs and porous Si (PS) templates are of interest (Bell et al., 1996; Seals et al., 2002; Aravamudhan et al., 2007). PS with specific dimensions is easily prepared by anodizing Si wafers with or without the assistance of a magnetic field. Au, Cu, Fe, Ni, and Co NWs have been deposited into PS templates via electrochemical routes, or chemical vapor deposition. These composites have potential in magnetic sensors and magneto-optic devices in integrated Si-based circuits, and detectors of spin-injection from ferromagnetic metals to Si. (Granitzer and Rumpf, 2010, 2011; Granitzer et al., 2013).

## Conclusion and outlook

The synthesis of metal NWs using hard templates was reviewed. AAO, MOs, and CNTs are often employed as hard templates, because their pore dimensions are controllable at the nanoscale. NWs prepared using hard templates do not require subsequent immobilizing or aligning, in contrast to those prepared using soft templates in solution. Table 1 shows the comparison of NWs obtained using the various hard templates. Commonly, AAO is employed to prepare NW arrays with high periodicity. MO produces NW arrays with smaller dimensions than ones produced with AAO. CNT brings about formation of isolated NWs covered with the CNT. TEPP and PS are sometimes used when the dimensions or composition of the templates are appropriate for specific applications. The NWs can be used without removing the template before application. For example, Co NW arrays in AAO are expected to be used as metamaterials due to their long-range high periodicity (Ye et al., 2003), and Fe, Co, or Ni NWs in CNT can be applied to medicine owing to the magnetic anisotropy and anti-corrosivity resulting from the protection of the NWs by a layer of graphite (Weissker et al., 2010). Metal NWs prepared using hard templates are compatible with large-scale production, so are suitable for industrial application.

**Table 1:** **Comparison of NWs obtained using various hard templates**.

**Templates**	**Metals**	**Diameters of NWs**	**Lengths of NWs**	**Arrayed/isolated**
AAO	Cu, Fe, Ni, Co, CoNi, Bi, Pd	5.5–450 nm	<100 μm	Arrayed
MO	Au, Pt, Ag, Cu, Co, Ni	2–15 nm	<4 μm	Arrayed
CNT	Pb, Bi, Y, Mg, Gd, Ti, Cr, Fe, Co, Ni, Cu, Zn, Mo, Pd, Ta, W, Dy, Yb, Au, Pt	7–100 nm	<70 μm	Isolated
TEPP	Au	~250 nm	<10 μm	Arrayed
PS	Au, Cu, Fe, Ni, Co	20–300 nm	<100 μm	Arrayed

### Conflict of interest statement

The authors declare that the research was conducted in the absence of any commercial or financial relationships that could be construed as a potential conflict of interest.
